# Patterns of acute poisoning for children during outbreak of Corona virus in Makkah region Saudi Arabia

**DOI:** 10.3389/fped.2023.1087095

**Published:** 2023-03-17

**Authors:** Bashayer Mohammed Althobaiti, Mahmoud Zaki El-Readi, Mohammad Althubiti, Yosra Zakariyya Alhindi, Abdullah R Alzahrani, Saeed S Al-Ghamdi, Nahla Ayoub, Bassem Refaat, Safaa Yehia Eid

**Affiliations:** ^1^ Department of Pharmacology and Toxicology, Faculty of Medicine, Umm Al-Qura University, Makkah, Saudi Arabia; ^2^ Saudi Toxicology Society, Umm Al-Qura University, Makkah, Saudi Arabia; ^3^ Department of Biochemistry, Faculty of Medicine, Umm Al-Qura University, Makkah, Saudi Arabia; ^4^ Biochemistry Department, Faculty of Pharmacy, Al-Azhar University, Assuit, Egypt; ^5^ Laboratory Medicine Department, Faculty of Applied Medical Sciences, Umm Al-Qura University, Makkah, Saudi Arabia

**Keywords:** patterns, acute, poisoning, children, outbreak, Corona virus, makkah

## Abstract

**Background:**

Poisoning occurs when a person is exposed to an external substance at a too high dose for them. It is possible for young children to be exposed to chemicals. Lungs, the heart, CNS, the digestive tract, and kidneys can be poisoned. In 2004, over 45,000 children and teenagers died from acute poisoning, representing 13% of all accidental poisoning deaths worldwide. Poisoning patterns vary by exposure type, age group, poison type, and dose.

**Aim:**

This study assessed the pattern of acute poisoning with drugs, chemicals, and natural toxins among children (<12 years old). The study was done in Makkah region and registered in the poison control center in Makkah, the forensic chemistry center in Haddah during 2020–2021.

**Methods:**

A retrospective cohort study was done on 122 children exposed to toxic substances in Makkah. The children were 12 years old and had good health for a maximum of one year. Stratified random sampling was used to divide cases into groups of similar poisons (pharmaceutical products, household products, plant envenomation, and animal envenomation). Then each group got a random samples. The data were analysed with SPSS software.

**Results:**

The mean age of children was 5.2 years, with 59% being boys. The mean temperature, pulse, systolic, diastolic, and respiratory rates were 36.77, 98.29, 109.1, 69.17, and 21.49. The most documented pharmaceutical products (200 mg) were carbamazepine (5 mg), methanol, risperidone (5 mg), propranolol (5 mg), and olanzapine (5 mg). The most common poison forms were tablets (42.6%), syrups (15.6%), capsules (13.9%), and solutions (13.1%). The most common poisoning routes were ingestion (82.8%), dermal (5.7%), injection (4.9%), and inhalation (6.6%). Accidental poisoning was 83%, with a 30-minute lag for 30.3% of children, and most (69.7%) occurred at home. Benzodiazepines were the most commonly used category class drug (18%), with normal pupils and an ECG of 85.2%. Sixty-seven percent had blood tests. Sickness was 9.48, and the positive result was 213.01. The most common presenting symptoms were GIT and neurological (23.8%). 31.1% had mild, moderate, or severe toxicity. Most cases (68%) were complex. 34.4% were intubated, 9.8% had repeated-dose-activated charcoal for enhanced elimination, and 27.8% were on IV fluids. Children with GIT, CVS, respiratory, dermal, and neurological symptoms had a higher percentage of severe toxicity (*p* < 0.05). Slight toxicity was associated with whole bowel irrigation, intubation for oxygen therapy, N-acetylcysteine or sedation, fluids, and phenytoin (*P* < 0.05). Complicated cases had a higher mean AST/IUL than non-complicated cases (75.5 vs. 20.08, *p* < 0.05). The level of toxicity did not correlate with the mean of all lab tests (*p* > 0.05). The age of the children correlated positively with their systolic BP (*r* = 0.22, *p* < 0.01).

**Conclusion:**

The results show how important it is to teach the public about poisoning and make rules for tracking and dealing with poisonings in Saudi Arabia.

## Introduction

Accidental poisonings take place when a person, typically a child, ingests a poisonous substance without intending to do so (as opposed to purposeful poisoning or overdosing) (1). Children are more likely to experience serious repercussions from poisoning due to the fact that their bodies are smaller, have a faster metabolic rate, and are less capable of neutralizing harmful substances (2). Children who are poisoned might experience psychological and physical repercussions over a long period of time, and the costs to society can be quite high (3).

Poisons ingested can be classified into medications (prescription or non-prescription), household items, and plants Their level of toxicity could be mild, moderate, or severe (4). Poisoning patterns vary depending on the type of exposure, age group, nature, and dose of the poison (5, 6).

Acute poisoning is a common occurrence in emergency rooms worldwide, necessitating extensive medical care and significant financial investment (7). A high number of acute poisoning cases were caused by drug poisoning. Natural poisons, such as toxic plants and animals and acute chemical poisonings in the home, are frequent, especially in children (8). There are various variations in the pattern and etiology of acute poisoning, even within the same geographical region (8).

The pattern and types of poisons vary depending on numerous factors such as demography, education, socioeconomic level, and local beliefs and practices in different parts of the world. As a result, each country needs its epidemiological surveillance to establish the scope and pattern of the disease so that preventative steps can be taken (9).

Knowing the overall trend of poisoning in a certain area can aid in identifying risk factors and enabling early discovery and treatment of such cases, lowering morbidity and mortality (8). Poisoning occurrences were treated differently depending on the patient's condition, the type of poisoning, and the length of exposure (10).

In Saudi Arabia (KSA), acute poisoning in children and adults has been reported in several Saudi cities, including Jeddah, Hafr Al Batin, Abha, and Al Riyadh (11). In Abha, there were 114 acute poisoning incidents in children between January 2000 and October 2003 (12). At King Khaled Hospital in the Al Majmaah region of Saudi Arabia, a study done in 2014 found that most instances were caused by animal envenomation (13). Alghadeer et al., 2018 found that most instances were asymptomatic, and most of the youngsters arrived at the hospital in under three hours (5).

Another perspective study conducted in Riyadh in 2019 found that toxic household goods were the most implicated substance class in children under the age of six (13), and recently in 2020, a study conducted at East Jeddah Hospital in Jeddah city found that unintentional poisoning occurred in 56.5% of recorded instances and 92.8% of incidents occurred at home (14).

This study aimed to assess the pattern of acute poisoning with drugs, chemicals, and natural toxins of children (≤12) in the Makkah region and registered in the poison control center in Makkah, Saudi Arabia. This study can provide additional information about the agents most commonly involved in poisoning in this region and prevention and management guidelines to avoid them.

## Subjects and methods

### Study design

A retrospective cohort study was done.

### Study population, setting and time frame

122 children with toxicity from the Children's Hospital in Makkah region of Saudi Arabia and registered in the poison control center in Makkah, forensic chemistry center in Haddah during 2020–2021 were included. The inclusion criteria were all children taking toxic substances any way route, of both genders, and children who live in Makkah. The exclusion criteria were forensic toxicology children with poisoning, adults, those that do not need medical management or recommendation, and children from regions other than Makkah.

### Sampling methodology

Stratified Random Sampling was done by dividing cases into groups according to the type of poisons with similar attributes (Pharmaceutical products-Household products-plant envenomation-animal envenomation). Then, from each group, a random sample was drawn. Children were classified into having mild, moderate, and severe toxicity as follows: (1) *Mild*; transient and spontaneously resolving symptoms, (2) *Moderate*; pronounced or prolonged symptoms, and (3) *Severe* or life-threatening symptoms. The sample size was calculated by this website (Qualtrics XM of sample size calculator). This equation was done at a confidence level of (95%) and a (5%) margin of error. The ideal sample size was (one hundred twenty-two).

### Ethical considerations

No issues regarding animal subjects. This study has had the approval of the research ethics committee at the Department of the deanship of postgraduate studies at Umm al Qura University in the Makkah region and the poison control center in Makkah forensic chemistry center. Written informed consent for the research was not required in accordance with National legislation and institutional requirements. No identifiable human images or data was present in the study.

### Data analysis

The data were analyzed using a statistical package for social sciences (SPSS) version 26. (Armonk, NY: IBM Corp.). Qualitative data were expressed as numbers and percentages to test the relationship between variables, and the Chi-squared test (*χ*2) was used. Quantitative data were expressed as mean and standard deviation (Mean ± SD), and non-parametric variables were tested using the Mann-Whitney (U) and Kruskal Wallis tests. Correlation analysis was performed using the Spearman's test, and a *p*-value of less than 0.05 was considered statistically significant.

### Results

The mean age of the studied children was 5.2 ± 3.74 years, and the mean BMI was 26.67 ± 24.86 kg/m^2,^ respectively. Of the children, 59% were males, and 58.2% had a Saudi nationality. 38.5% of children had mild toxicity, while 31.1% and 30.3% had moderate and severe toxicity. And most of the cases (68%) were complicated. However, 32% of cases were not complicated. For 30.3% of children, the duration since poisoning was 30 min, 26.2% was one hour, 21.3% was 2 h, 8.2% was 3–12 h, and for 13.9%, the duration was 12 h. For most children (69.7%), the poisoning happened at home.

Table 1 shows that the most common documented pharmaceutical products were Depakine, Olanzapine (5 mg), Cannabinoids, Carbamazepine, Hydrogen peroxide (6%), Methanol, Risperidone lorazepam, Propranolol, and valproate sodium (200 mg). A non-significant relationship was found between the level of toxicity and the taken pharmaceutical products (*p* > 0.05).

**Table 1:** Relationship between pharmaceutical products and the level of toxicity.

Variable	Total	Toxicity level			*χ*2	*p*-value
	No. (%)	Mild	Moderate	Severe		
Pharmaceutical products						
− Depakine	1 (0.8)	1 (100)	0 (0.0)	0 (0.0)		
− Olanzapine 5 mg	1 (0.8)	0 (0.0)	1 (100)	0 (0.0)		
− Cannabinoids	1 (0.8)	1 (100)	0 (0.0)	0 (0.0)	13.8	0.464
− Carbamazepine	1 (0.8)	0 (0.0)	0 (0.0)	1 (100)		
− Hydrogen peroxide 6%	1 (0.8)	1 (100)	0 (0.0)	0 (0.0)		
− Methanol	1 (0.8)	0 (0.0)	0 (0.0)	1 (100)		
− Risperidone	1 (0.8)	0 (0.0)	1 (100)	0 (0.0)		
− Lorazepam	1 (0.8)	0 (0.0)	1 (100)	0 (0.0)		
− Propranolol	1 (0.8)	1 (100)	0 (0.0)	0 (0.0)		
− Valproate-Na (200 mg)	1 (0.8)	0 (0.0)	1 (100)	0 (0.0)		
Dosage form						
− Capsule	17 (13.9)	6 (35.3)	8 (47.1)	3 (17.6)	6.1	0.412
− Chewable tablet	1 (0.8)	1 (100)	0 (0.0)	0 (0.0)		
− Gas	2 (1.6)	0 (0.0)	1 (50)	1 (50)		
− Injection	6 (4.9)	2 (33.3)	3 (50)	1 (176.7)		
− Mouth wash	1 (0.8)	0 (0.0)	0 (0.0)	1 (100)		
− Powder	3 (4.5)	0 (0.0)	0 (0.0)	3 (100)		
− Solution	16 (13.1)	5 (31.3)	5 (31.3)	6 (37.5)		
− Suppository	1 (0.8)	1 (100)	0 (0.0)	0 (0.0)		
− Suspension	3 (2.5)	2 (66.7)	1 (33.3)	0 (0.0)		
− Syrup	19 (15.6)	5 (26.3)	5 (26.3)	9 (47.4)		
− Tablet	52 (42.6)	25 (48.1)	14 (26.9)	13 (25)		
− Inhalation	1 (0.8)	1 (100)	0 (0.0)	0 (0.0)		

Table 2 shows that the most common category class drug used were Benzodiazepine (18%), followed by Analgesic non opioid (15.6%), senna leaves rhubarb root (7.4%), and alcohol or NSAID (4.1%). A non-significant relationship was found between the level of toxicity and major category class drugs used (*p* > 0.05).

**Table 2:** Relationship between major drug's class and level of toxicity.

Major drug class	Total	Toxicity level			*χ*2	*p*-value
	No. (%)	Mild	Moderate	Severe		
− 3,4-Methyl one deoxymethamphetamine	1 (0.8)	1 (100)	0 (0.0)	0 (0.0)		
− Aerosol spray	1 (0.8)	0 (0.0)	0 (0.0)	1 (100)		
− Alcohol	5 (4.1)	2 (40)	2 (40)	1 (20)		
− Analgesic	2 (1.6)	2 (100)	0 (0.0)	0 (0.0)	84.74	0.145
− Analgesic non-opioid	19 (15.6)	10 (52.6)	8 (42.1)	1 (5.3)		
− Anticholinergic	1 (0.8)	0 (0.0)	1 (100)	0 (0.0)		
− Anticonvulsants	2 (1.6)	0 (0.0)	1 (50)	1 (50)		
− Antidiabetic drug	2 (1.6)	1 (50)	1 (50)	0 (0.0)		
− Antidepressant	2 (1.6)	0 (0.0)	2 (100)	0 (0.0)		
− Antiepileptic, anticonvulsant	2 (1.6)	0 (0.0)	1 (50)	1 (50)		
− Antihistamine	3 (2.5)	2 (50)	1 (50)	0 (0.0)		
− Antipsychotic and antihypertensive	1 (0.8)	1 (100)	0 (0.0)	0 (0.0)		
− Antipyretic	1 (0.8)	1 (100)	0 (0.0)	0 (0.0)		
− Atypical antipsychotics	3 (2.5)	0 (0.0)	1 (33.3)	2 (66.7)		
− Benzodiazepine	22 (18)	8 (36.4)	10 (45.4)	4 (18.2)		
− Cannabis	3 (2.5)	0 (0.0)	0 (0.0)	3 (100)		
− CNS stimulant	1 (0.8)	0 (0.0)	1 (100)	0 (0.0)		
− CNS stimulants, Hallucinogens	1 (0.8)	0 (0.0)	0 (0.0)	1 (100)		
− Corrosive agent	3 (2.5)	1 (33.3)	0 (0.0)	2 (66.7)		
− Detergent	1 (0.8)	0 (0.0)	1 (100)	0 (0.0)		
− General anesthetics	1 (0.8)	1 (100)	0 (0.0)	0 (0.0)		
− Glycopeptide antibiotics	1 (0.8)	0 (0.0)	1 (100)	0 (0.0)		
− Hallucinogens	1 (0.8)	1 (100)	0 (0.0)	0 (0.0)		
− Heavy metal	1 (0.8)	0 (0.0)	1 (100)	0 (0.0)		
− Hydrogen peroxide	1 (0.8)	1 (100)	0 (0.0)	0 (0.0)		
− NSAID	5 (4.1)	1 (20)	3 (60)	1 (20)		
− Opioid	4 (3.3)	2 (50)	2 (50)	0 (0.0)		
− Phenethylamine	7 (5.7)	0 (0.0)	2 (29)	5 (71)		
− Proton pump inhibitors	1 (0.8)	1 (100)	0 (0.0)	0 (0.0)		
− Psychoactive drugs	1 (0.8)	1 (100)	0 (0.0)	0 (0.0)		
− Second-generation antipsychotics	3 (2.5)	0 (0.0)	2 (66.7)	1 (33.3)		
− Sedative-hypnotics	2 (1.6)	0 (0.0)	1 (50)	1 (50)		
− Selective serotonin reuptake inhibitors	3 (2.5)	0 (0.0)	2 (66.7)	1 (33.3)		
− Senna leaves Rhubarb Root Powder	9 (7.4)	3 (33.3)	3 (33.3)	3 (33.3)		
− Supplement food	2 (1.6)	2 (100)	0 (0.0)	0 (0.0)		
− Volatile substances	4 (3.3)	2 (50)	1 (25)	1 (25)		

Table 3 shows that the most common presenting symptoms were both GIT and neurological symptoms (23.8%), followed by only neurological symptoms (16.4%) and only GIT symptoms (13.1%). The table shows that children who were presented with (CVS, dermal and neurological symptoms) or (CVS and neurological symptoms) or (GIT, CVS, respiratory, dermal and neurological symptoms) or (GIT, CVS, dermal and neurological) or (Respiratory, dermal and neurological) had a significantly higher 100% of having severe toxicity (*p* < 0.05).

**Table 3:** Relationship between presenting symptoms and level of toxicity.

Symptoms	Total	Toxicity level			*χ*2	*p*-value
	No. (%)	Mild	Moderate	Severe		
− Only GIT symptoms	20 (13.1)	14 (62.5)	6 (37.5)	0 (0.0)	70.23	0.004
− GIT, CVS, respiratory, dermal, and neurological	2 (1.6)	0 (0.0)	0 (0.0)	2 (100)		
− GIT, CVS, respiratory, and neurological	2 (1.6)	0 (0.0)	2 (100)	0 (0.0)		
− GIT, CVS, dermal, and neurological	2 (1.6)	0 (0.0)	0 (0.0)	2 (100)		
− GIT, CVS, and neurological	1 (0.8)	0 (0.0)	1 (100)	0 (0.0)		
− GIT and respiratory	1 (0.8)	1 (100)	0 (0.0)	0 (0.0)		
− GIT, respiratory, and dermal	2 (1.6)	0 (0.0)	2 (100)	0 (0.0)		
− GIT, respiratory, dermal, and neurological	5 (4.1)	1 (20)	4 (80)	0 (0.0)		
− GIT, respiratory and neurological	12 (9.8)	5 (41.7)	3 (25)	4 (33.3)		
− GIT and dermal symptoms	3 (2.5)	2 (66.7)	0 (0.0)	1 (33.3)		
− GIT, dermal, and neurological	1 (0.8)	1 (100)	0 (0.0)	0 (0.0)		
− GIT and neurological	29 (23.8)	16 (55.2)	8 (27.6)	5 (17.2)		
− Only CVS	1 (0.8)	0 (0.0)	1 (100)	0 (0.0)		
− CVS, respiratory, and neurological	3 (2.5)	1 (33.3)	0 (0.0)	2 (66.7)		
− CVS, dermal, and neurological	2 (1.6)	0 (0.0)	0 (0.0)	2 (100)		
− CVS and neurological symptoms	1 (0.8)	0 (0.0)	0 (0.0)	1 (100)		
− Only respiratory	5 (4.1)	1 (20)	1 (20)	3 (60)		
− Respiratory, dermal, and neurological	1 (0.8)	0 (0.0)	0 (0.0)	1 (100)		
− Respiratory and neurological	5 (4.1)	0 (0.0)	2 (40)	3 (60)		
− Dermal and neurological	3 (2.5)	0 (0.0)	1 (33.3)	2 (66.7)		
− Only neurological symptoms	21 (16.4)	5 (25)	6 (30)	10 (45)		

Table 4 shows that almost one-quarter of children had activated charcoal (25.4%), 8.2% received N-acetylcysteine, (34.4%) were intubated, and (9.8%) had repeated-dose-activated charcoal for enhancement elimination, 9% had IV fluids as pre-hospital management, and 3.3% were ventilated. Almost one-third of children were on IV fluid (27.8%), 4.1% were on Activated charcoal or N-acetylcysteine or antibiotics, 7.4% were on Flumazenil, and 1.6% were on omeprazole injection. Children who had whole bowel irrigation were intubated for oxygen therapy and had N-acetylcysteine or sedation, fluids, and phenytoin had a significantly higher % of having severe toxicity (*p* < 0.05).

**Table 4:** Relationship between GIT decontamination, antidote, oxygen therapy, enhancement elimination, pre-hospital management, and current medications and level of toxicity.

Variable	Total	Toxicity level			χ2	*p*-value
	No. (%)	Mild	Moderate	Severe		
GIT decontamination						
− No GIT decontamination	71 (58.2)	19 (26.8)	22 (31)	30 (42.3)		
− Activated charcoal	31 (25.4)	20 (64.5)	8 (25.8)	3 (9.7)	23.17	0.01
− Dilution	3 (2.5)	2 (66.7)	1 (33.3)	0 (0.0)		
− Gastric lavage	15 (12.3)	5 (33.3)	7 (46.7)	3 (20)		
− Ipecac syrup	1 (0.8)	1 (100)	0 (0.0)	0 (0.0)		
− Whole bowel irrigation	1 (0.8)	0 (0.0)	0 (0.0)	1 (100)		
Antidote						
− No antidote	97 (79.5)	42 (43.3)	28 (28.9)	27 (27.8)	18.69	0.177
− Atropine	1 (0.8)	0 (0.0)	1 (100)	0 (0.0)		
− Deferoxamine	1 (0.8)	1 (100)	0 (0.0)	0 (0.0)		
− Flumazenil	7 (5.7)	1 (14.3)	1 (14.3)	5 (71.4)		
− N-Acetylcysteine	10 (8.2)	2 (20)	5 (50)	3 (30)		
− Naloxone	4 (3.3)	0 (0.0)	2 (50)	2 (50)		
− Physostigmine	1 (0.8)	0 (0.0)	1 (100)	0 (0.0)		
− Thiamin	1 (0.8)	1 (100)	0 (0.0)	0 (0.0)		
Oxygen therapy						
− No oxygen therapy	80 (65.6)	39 (48.4)	22 (27.5)	19 (23.8)	10.61	0.005
− Intubated	42 (34.4)	8 (19)	16 (38.1)	18 (42.9)		
Enhancement Elimination						
− No enhancement elimination	103 (84.4)	40 (38.8)	31 (30.1)	32 (31.1)		
− Forced acidic diuresis	2 (1.6)	0 (0.0)	2 (100)	0 (0.0)		
− Forced alkaline diuresis	3 (2.5)	1 (33.3)	1 (33.3)	1 (33.3)	9.68	0.468
− Hemodialysis	1 (0.8)	0 (0.0)	0 (0.0)	1 (100)		
− Hemoperfusion	1 (0.8)	0 (0.0)	1 (100)	0 (0.0)		
− Repeated-dose activated charcoal	12 (9.8)	6 (50)	3 (25)	3 (25)		
Pre-hospital Management						
− No pre-hospital management	97 (79.5)	43 (44.3)	24 (24.7)	30 (30.9)		
− Activated charcoal	1 (0.8)	1 (100)	0 (0.0)	0 (0.0)		
− Atropine-Diazepam	1 (0.8)	0 (0.0)	1 (100)	0 (0.0)	37.45	0.039
− Diazepam, phenytoin and phenobarbitone	1 (0.8)	0 (0.0)	0 (0.0)	1 (100)		
− Drink juice	1 (0.8)	0 (0.0)	1 (100)	0 (0.0)		
− Drink water	2 (1.6)	0 (0.0)	2 (100)	0 (0.0)		
− Flumazenil	1 (0.8)	0 (0.0)	0 (0.0)	1 (100)		
− IV fluid	10 (8.2)	1 (10)	8 (80)	1 (10)		
− IV fluid and vitamin	1 (0.8)	0 (0.0)	1 (100)	0 (0.0)		
− Keppra 500 mg	1 (0.8)	1 (10)	0 (0.0)	0 (0.0		
− N-acetylcysteine	1 (0.8)	0 (0.0)	0 (0.0)	1 (100)		
− Sedation, fluids, and phenytoin	1 (0.8)	1 (100)	0 (0.0)	1 (100)		
− Ventilated	4 (3.3)	1 (25)	1 (25)	2 (50)		
Current medications						
− No current medications	48 (39.3)	18 (37.5)	14 (29.2)	16 (33.3)		
− Activated charcoal	5 (4.1)	2 (40)	1 (20)	2 (40)	51.84	0.326
− Acyclovir and ceftriaxone	3 (2.5)	1 (33.3)	1 (33.3)	1 (33.3)		
− Antibiotic	5 (4.1)	3 (60)	0 (0.0)	2 (40)		
− Ceftriaxone, acyclovir, and vancomycin	1 (0.8)	0 (0.0)	0 (0.0)	1 (100)		
− Fentanyl and midazolam	1 (0.8)	0 (0.0)	0 (0.0)	1 (100)		
− Flumazenil	1 (0.8)	1 (100)	0 (0.0)	0 (0.0)		
− IV fluid	32 (26.2)	12 (37.5)	15 (46.9)	5 (15.6)		
− IV fluid, thiamin	1 (0.8)	1 (100)	0 (0.0)	0 (0.0)		
− IV fluid, vitamin	1 (0.8)	1 (100)	0 (0.0)	0 (0.0)		
− Laxative	2 (1.6)	1 (50)	1 (50)	0 (0.0)		
− Mannitol, dezocine	1 (0.8)	0 (0.0)	0 (0.0)	1 (100)		
− Mechanical ventilation	1 (0.8)	1 (100)	0 (0.0)	0 (0.0)		
− Methylprednisolone	1 (0.8)	0 (0.0)	1 (100)	0 (0.0)		
− N-acetylcysteine	5 (4.1)	1 (20)	2 (40)	2 (40)		
− NAD	2 (1.6)	0 (0.0)	2 (100)	0 (0.0)		
− Naloxone	1 (0.8)	1 (100)	0 (0.0)	0 (0.0)		
− Observation	2 (1.6)	1 (50)	1 (50)	0 (0.0)		
− Omeprazole injection	2 (1.6)	0 (0.0)	0 (0.0)	2 (100)		
− Pain killer, antibiotic	1 (0.8)	1 (100)	0 (0.0)	0 (0.0)		
− Supportive treatment	1 (0.8)	0 (0.0)	1 (0.0)	0 (0.0)		
− Tazocine and IV fluid	1 (0.8)	0 (0.0)	0 (0.0)	1 (100)		
− Vancomycin	1 (0.8)	1 (100)	0 (0.0)	0 (0.0)		
− Ventolin, Methylprednisolone, phenytoin, vancomycin, and acyclovir	1 (0.8)	1 (100)	0 (0.0)	0 (0.0)		
− Vitamin	2 (1.6)	2 (100)	0 (0.0)	0 (0.0)		

Table 5 demonstrated a non-significant relationship between the level of toxicity among studied children and their age, height, weight BM, gender, nationality, pharmaceutical products, or poison forms (*p* > 0.05).

**Table 5:** Relationship between level of the toxicity and the children's characters, weight, BMI. pharmaceutical products and poison forms (*n *= 122).

Variable	Toxicity level			*χ*2-one-way Anova	*p*-value
	Mild	Moderate	Severe		
Age	5.63 ± 3.93	4.86 ± 3.4	5.18 3.65	2^a^	0.652
Height	10,308 ± 31.73	96.4 ± 25.99	104.1 ± 25.12	2^a^	0.386
Weight	27.38 ± 14.6	22.03 ± 9.49	23.59 ± 9.56	2^a^	0.398
BMI	29.66 ± 33.29	27.11 ± 23.61	22.43 ± 7.6	2^a^	0.191
Gender					
− Female	20 (40)	18 (36)	12 (24)	1.8	0.405
− Male	27 (37.5)	20 (27.8)	25 (34.7)		
Nationality					
− Non-Saudi	17 (33.3)	15 (29.4)	19 (37.3)	2.08	0.353
− Saudi	30 (42.3)	23 (32.4)	18 (25.4)		
Pharmaceutical products (1)					
− Depakin	1 (100)	0 (0.0)	0 (0.0)		
− Olanzapine 5 mg	0 (0.0)	1 (100)	0 (0.0)	13.8	0.464
− Cannabinoids	1 (100)	0 (0.0)	0 (0.0)		
− Carbamazepine	0 (0.0)	0 (0.0)	1 (100)		
− Hydrogen peroxide 6%	1 (100)	0 (0.0)	0 (0.0)		
− Methanol	0 (0.0)	0 (0.0)	1 (100)		
− Two pharmaceutical products (1)	44 (38.3)	36 (31.3)	35 (30.4)		
− Risperidone	0 (0.0)	1 (100)	0 (0.0)		
Pharmaceutical products (2)					
− Lorazepam	0 (0.00	1 (100)	0 (0.0)		
− Two pharmaceutical products (2)	46 (38.7)	36 (30.3)	37 (31.1)	6.07	0.415
− Propranolol	1 (100)	0 (0.0)	0 (0.0)		
− Valproate sodium 200 mg	0 (0.0)	1 (100)	0 (0.0)		
Poison forms					
− Capsule	0 (0.0)	1 (100)	0 (0.0)		
− Inhalation	1 (100)	0 (0.0)	0 (0.0)	6.1	0.412
− Two poison forms	46 (38.7)	37 (31.1)	36 (30.3)		
− Tablet	0 (0.0)	0 (0.0)	1 (100)		

^a^ N.B.:  = Kruskal Wallis test.

Table 6 shows that the most common poison forms were tablets (42.6%), syrups (15.6%), capsules (13.9%), solutions (13.1%) poisoning by pharmaceutical products (80.3%), household products accounts (12.3%), plant envenomation (5.7%,) and by animal envenomation (1.6%) as for the route of poisoning, ingestion accounts for (82.8%), dermal route (5.7%), injection (4.9%), inhalation (6.6%). Accidental poisoning was (83%), and intentional poisoning accounts for (39%) of cases. A non-significant relationship was found between the level of toxicity and the taken pharmaceutical products, poison forms, route or mode of poisoning (*p* => 0.05).

**Table 6:** Relationship between level of toxicity and poison forms, route and mode of poisoning, time of exposure, and place of poisoning (No.:122).

Variable	Toxicity level			*χ*2	*p*-value
	Mild	Moderate	Severe		
**Type of poisons**	No. (%)	No. (%)	No. (%)		
**All types**					
Animal envenomation	1 (50)	1 (50)	0 (0.0)	4.74	0.577
Household products	3 (20)	5 (33.3)	7 (46.7)		
Pharmaceutical products	40 (40.8)	29 (29.6)	29 (29.6)		
Plant envenomation	3 (42.9)	3 (42.9)	1 (14.3)		
Pharmaceutical products					
No	7 (29.2)	9 (37.5)	8 (33.3)	1.43	0.487
Yes	40 (40.8)	29 (29.6)	29 (29.6)		
Household products					
No	44 (41.9)	33 (31.4)	30 (26.7)	5.58	0.061
Yes	3 (20)	5 (33.3)	7 (46.7)		
Plant envenomation					
No	44 (38.3)	35 (30.4)	36 (31.3)	2.74	0.254
Yes	3 (42.9)	3 (42.9)	1 (14.3)		
Animal envenomation					
No	46 (38.3)	37 (30.8)	37 (30.8)	0.91	0.632
Yes	1 (50)	1 (50)	0 (0.0)		
**Route of poisons**					
Ingestion					
No	6 (28.6)	8 (38.1)	7 (33.3)	2.62	0.269
Yes	41 (40.6)	30 (29.7)	30 (29.7)		
Dermal					
No	45 (39.1)	36 (31.3)	34 (29.6)	2.02	0.363
Yes	2 (28.6)	2 (28.6)	3 (42.8)		
Injection					
No	46 (39.6)	35 (30.2)	35 (30.2)	1.52	0.467
Yes	1 (16.7)	3 (50)	2 (33.3)		
Inhalation					
No	44 (38.6)	35 (30.7)	35 (30.7)	2.49	0.288
Yes	3 (37.5)	3 (37.5)	2 (25)		
**Mode of poisoning**					
Accidental					
No	16 (41)	12 (30.8)	11 (28.2)	0.18	0.914
Yes	31 (37.3)	26 (31.3)	26 (31.3)		
Intentional					
No	31 (37.3)	26 (31.3)	26 (31.3)	0.48	0.787
Yes	16 (41)	12 (31.8)	11 (28.2)		
**Time of exposure**					
30 min					
No	32 (37.6)	26 (30.6)	27 (31.8)	0.27	0.872
Yes	15 (40.5)	12 (324)	10 (27)		
One hour					
No	31 (34.4)	30 (33.3)	29 (32.2)	241	0.299
Yes	16 (50)	8 (25)	8 (25)		
Two hour					
No	39 (40.6)	28 (29.2)	29 (30.2)	1.08	0.581
Yes	8 (30.8)	10 (38.5)	8 (30.80		
3-12 h					
No	46 (41.1)	35 (31.3)	31 (27.7)	5.46	0.065
Yes	1 (10)	3 (30)	6 (60)		
More than 12 h					
No	42 (40)	31 (29.5)	32 (30.5)	1.06	0.586
Yes	5 (29.4)	7 (41.2)	5 (29.4)		
**Place of poisoning**					
Home					
No	15 (40.5)	10 (27)	12 (32.4)	0.42	0.809
Yes	32 (37.6)	28 (32.9)	25 (29.4)		
Hospital					
No	44 (37.6)	36 (30.8)	37 (31.6)	2.33	0.311
Yes	3 (60)	2 (40)	0 (0.0)		
Outside home					
No	36 (39.6)	30 (33)	25 (27.5)	1.44	0.486
Yes	11 (35.5)	8 (25.8)	12 (38.7)		

Table 7 shows that complicated cases had a significantly higher mean level of AST (IU/l) than non-complicated cases (75.5 vs. 20.08 (*p* < 0.05). On the other hand, a non-significant relationship was found between the presence of complicated cases and all other laboratory test results of the studied children (*p* > 0.05).

**Table 7:** Relationship between complicated cases and laboratory results (No.:122).

Variable	Complications		U- test	*p*-value
	Complicated cases	Non-complicated case		
	Mean ± SD	Mean ± SD		
pH	7.34 ± 0.14	7.35 ± 0.09	0.29	0.771
HCO3 (mmol)	22.43 ± 5.39	23.16 ± 4.46	0.55	0.581
PaO2 (mmHg)	83.2 ± 17.3	80.08 ± 16.75	0.91	0.359
So2	93.48 ± 6.21	94.14 ± 7.17	0.65	0.512
Paco2 (mmHg)	39.49 ± 13.35	38.31 ± 9.06	0.09	0.923
Na (mEqL)	138.38 ± 6.24	138.51 ± 4.18	0.27	0.784
K (mEqL)	4.31 ± 0.7	4.08 ± 0.06	1.76	0.078
Cl (mEqL)	103.07 ± 10.63	101.21 ± 5.76	0.89	0.369
Mg (mg/dl)	1.7 ± 0.46	1.73 ± 0.41	0.63	0.527
Cholinesterase level (IUL)	3053.38 ± 2942.96	3019.71 ± 2197.65	0.52	0.599
ALT (IUL)	68.66 ± 162.63	30.01 ± 9.13	0.6	0.549
AST (IUL)	75.5 ± 216.05	20.08 ± 13.08	2.8	**0** **.** **005**
S.bilirubin (mg/dl)	3.39 ± 14.79	0.95 ± 1.07	0.25	0.8
RBCs (Cell/ml)	4.65 ± 0.52	4.86 ± 0.99	1.12	0.259
Hb (gm/dl)	13.28 ± 2.06	13.67 ± 2.46	0.53	0.594
WBCs (Cell/ml)	12,793.84 ± 26,637.39	19,314.81 ± 45,475.02	1.2	0.229
Urea (mg/dl)	30.08 ± 9.96	31.9 ± 8.84	0.96	0.336
BUN (mg/dl)	18 ± 39.31	11.74 ± 5.8	0.34	0.733
INR	1.11 ± 0.26	1.09 ± 0.21	1.31	0.19

## Discussion

This study aimed to assess and analyse the patterns of acute poisoning cases with drugs, chemicals, and natural toxins for children in the Makkah region of Saudi Arabia registered in the Makkah poison control center and the Haddah forensic chemistry center.

In this study, male children accounted for 59% of the affected cases compared to 41% females. And the mean age was 5.2 ± 3.74 years. A previous Saudi study done in 2018 in Riyadh city found a prevalence of 49.7% among females, and the mean age of children was 2.7 ± 2.1 years (8).

Between 2010 and 2016, a review study conducted in the Riyadh region found that more than half of poisoning cases (62%) happened in youngsters under two (5). Another study conducted in the Jeddah region discovered that most cases occurred in male children (1), which was comparable with international studies (15, 16). Previously, the study that conducted in Sri Lanka reported that the majority of children who ingested poisons were under the age of five years (16). The WHO also reported an overall higher rate of poisoning in boys than girls in different world regions (15).

Paracetamol was shown to be the most dangerous pharmaceutical product (94%), followed by other pharmaceuticals such as Depakine, Olanzapine 5 mg, and Risperidone lorazepam. In the present study, the most common category class drug used were Benzodiazepine (18%), followed by analgesic non opioid (15.6%), senna leaves rhubarb root (7.4%), and alcohol or NSAID (4.1%). A study conducted in Jeddah in 2020 found that ingested medicines were the leading cause of acute poisoning (73.9%), a finding that was consistent with a previous study (14) in which medicinal products were the leading cause of poisoning. Previously, the most frequently involved drug class was weak analgesics dominated by paracetamol (*n* = 91, 35%), followed by opioids and benzodiazepines (17).

Many reports, especially from Saudi Arabia, support this finding, highlighting medicine's role in self-poisoning (5, 18). The drug administration providing a reason this to delivering medication in envelopes rather than child-resistant containers, easy access to drugs without prescriptions, and irresponsible home drug storage (19). Furthermore, Saudi Arabia's higher rating for unintended drug poisoning may be related to Saudi families' habit of storing unused prescriptions for future usage (5). The ready availability of medications and chemicals in various forms at home and a lack of parental monitoring to keep these materials in a safe place and out of reach of children were the most common causes of childhood poisoning.

In the present work, it was observed that the most common dosage forms were tablets (41.8%). According to a previous study conducted in Abha (20), tablets' most common poison types (19). The present work found that ingestion was the most common rote of poisoning (93.4%), followed by cutaneous poisoning (13.9%), inhalation (9.8%), and injection (9.8%). This result agrees with previous studies done in KSA (5, 20).

This work observed that accidental poisoning accounted for (68%) of cases compared to 31.1% for intentional poisoning. The same result was found in a previous study done in KSA (1, 8) and others (21). The study showed that for (30.3%) of children, the time of exposure was 30 min, for (26.2%) it was one hour, and for (21.3%) it was 2 h. A study done in India found that the time interval between exposure to poison and admission to the hospital was less than 3 h in 73 (35.96%) cases, 3 to 6 h in 92 (45.32%) cases, and more than 6 h in 38 (18.71%) cases (22). Another study in Iran discovered that the average time from incident to hospitalization was 144.3171 min (23). The poisoning occurred at home for the majority of the youngsters (69.7%). The same result was revealed from other studies (1, 21).

In the present study, most symptoms appear as GIT symptoms. In the study done in Majmaah, 25.6% of children had GIT symptoms (8). In Abha, the most common symptoms of poisoning were nausea, vomiting (40.4%), and (16.7%) abdominal pain (18). In an Iranian study, 28.2 percent of youngsters experienced gastrointestinal symptoms (23).

In the current study, 38.5 percent of children experienced mild toxicity, whereas 31.1% and 30.3% had moderate and severe toxicity, respectively. Furthermore, most cases (68%) were complicated. However, 32% of cases were uncomplicated. The high prevalence of mild cases was found in previous studies (1, 16, 24).

Almost one-quarter of children had activated charcoal (25.4%), (12.3%) had gastric lavage, and (2.5%) had dilution. Activated charcoal was found to limit the absorption of various toxins and medications in the stomach and intestine, including antipsychotics, antiepileptics, and salicylates (5, 25). Clinical trials have shown that multi-dose administration of activated charcoal can prevent severe intoxication from carbamazepine, quinine, phenobarbital, and theophylline (26).

In a previous study, only 6% of cases received a specific antidote, which could be attributed to the short time between poisoning and arrival at the hospital (25). Approximately 28% of children were treated by gut decontamination with activated charcoal in Riyadh, and only 1.8% were given specific antidotes (5).

The limitation of this study was having a retrospective study design and singular location. Incomplete and missing data may further affect the study's findings' generalization.

## Conclusion

Based on the present study results, acute poisoning among children is a major public health issue in Saudi Arabia, so implementing a national policy for adequate and prompt management will result in a favorable outcome. In addition, children's exposure to harmful chemicals necessitates more attention, particularly among families, to raise their awareness of safety requirements within the home. Future studies are needed to clarify the role of various factors involved in childhood poisoning.

## Limitations

Due to its retrospective design and single-center setting, the study has limited general validity. The small sample size may also reduce the study's generalizability; therefore, we intend to conduct additional research involving multiple centres in different geographic regions.

## Data Availability

The raw data supporting the conclusions of this article will be made available by the authors, without undue reservation.
